# Four microRNAs Signature for Survival Prognosis in Colon Cancer using TCGA Data

**DOI:** 10.1038/srep38306

**Published:** 2016-12-15

**Authors:** Jian Xu, Jian Zhao, Rui Zhang

**Affiliations:** 1 Department of Colorectal Surgery, Cancer Hospital of China Medical University, Liaoning Cancer Hospital & Institute, Shenyang, People’s Republic of China.

## Abstract

This study aims to develop microRNA expression signature for colon cancer survival prognosis based on the Cancer Genomic Common database. miRNAs levels between colon cancer and non-cancer tissues were screened by t-test (p < 0.05). Kaplan-Meier survival method was used to discriminate survival significant miRNAs, followed by miRNAs index accumulation to power the miRNAs-survival reliability. In the end, we test the selected miRNAs in HT126 colon cancer cells to validate its anti-cancer effect. The study identified a 84-miRNAs signature. Of the above 84 miRNAs, we got four miRNAs which were survival associated by using ROC curve method and Kaplan-Meier survival method (p < 0.001). The result showed that low risk group had quite a low death rate, the survival rate was over 80%. The high risk group had survival rate lower than 20%, which was also extremely lower than the overall survival rate. In the HT126 cells study, cell growth assay showed miR-130a sponge inhibited colon cancer cells growth and sensitized the anti-cancer drug effect of 5-FU to blocked cancer cell growth. We developed a prognostic 4-microRNA expression signature for colon cancer patient survival, and validated miR-130a sponge could sensitized 5-FU anti-cancer effect.

Colon cancer is the third most commonly diagnosed cancer in males and the second in females, and the fourth greatest cause of cancer-related deaths worldwide^1^. Nowadays, tumor screening methods include the guaiac-based fecal occult blood test [FOBT], flexible sigmoidoscopy, stool DNA test, computed tomography [CT] colonography, double-contrast barium enema, and colonoscopy. Of these screening options, prognostic survival markers of patients are still under development. Hence, the identification of novel markers, which could indicate high risk or low risk in survival, would greatly optimize the use of therapies and benefit patients. Recently, prognostic microRNAs (miRNAs or miRs) expression signatures have been developed in cancers, and miRNAs signature has been realized as important epigenetic changes in cancer development and therapy^2^.

miRNAs are short 20–22 bp nucleotide, non-coding RNAs which play key roles in biological function. The abnormal expression level of miRNAs is realized as an important issue in cancer development. Therefore, miRNAs therapy is becoming a bright target^3^. These small molecules regulate gene expression through binding to the target mRNA, which influence mRNA stability or suppress translation^4^. It was reported that miRNAs were stable, even in formalin-fixed paraffin-embedded (FFPE) samples. Therefore, miRNAs analysis will be not affected by storage time in tissue samples^5^. Basically, miRNAs regulate the expression of more than 30% of human genes^6^.

It was reported that several miRNAs markers were identified in caners, such as head and neck squamous cell carcinomas^7^, six-miRNAs signature in bile duct cancer prediction^8^, and 5-miRNAs prognosis in glioma^9^. However, as to the smaller patient number or limited miRNAs number, or different miRNAs-chip platform in colon cancer study, studies lacked a normalized standard. Therefore, a larger patient cohort and normal controls as well as a standard protocol for more specific prognostic classifiers are warranted.

In the current study, we employed a large cohort of colon cancer patients to explore miRNAs expression signature for survival prognosis. We study prognostic value of miRNAs expression in colon cancer, with the aim of developing a multi-miRNAs prognostic expression signature. In this end, we assessed the expression of 1046 miRNAs in 467 patients from Genomic Data Commons Data Portal (https://gdc-portal.nci.nih.gov/). Additionally, we investigated the association of miRNAs expression index with the survival time, and ranked the risk index in colon cancer.

## Materials and Methods

### Patient cohort and miRNAs data

The results shown here were wholly based upon data generated by the TCGA Research Network: https://gdc.cancer.gov/. The dataset acquired above contained 1046 noted miRNAs expression data. The downloaded clinical data were matched to the miRNAs expression profile. Therefore, some patients were excluded, such as those missing miRNAs expression level, or those without follow up day, or those without most clinical information.

### Screening of differentially expressed miRNAs and Hierarchical clustering

Differentially expressed miRNAs between cancer and non-cancer tissues of colon cancer were screened by t-test in Excel file (p < 0.05). Multi-experimental viewer software was used to get hierarchical clustering map (choose hierarchical clustering method). The hierarchical clustering analysis guaranteed cancer and non-cancer specimens were correctly classified by miRNAs levels.

### Selection of cutoff score for the Kaplan-Meier survival analysis

We selected the cutoff scores based on receiver operating characteristic(ROC) curve analysis^10^. At each miRNAs expression level, the sensitivity and specificity for each outcome was plotted, and thus an ROC curve was generated. The highest score with both maximum sensitivity and specificity on the curve was selected as the cutoff point. The data was dichotomized into high level and low level groups, followed by the Kaplan-Meier survival analysis to determine having or not having clinical outcome^11^. To use ROC curve analysis, the clinical outcome and the miRNAs signature index were dichotomized: dead and alive in the follow-up data as clinical outcome, high risk and low risk in the index. Kaplan-Meier survival method was used to compare the different of two groups. ROC curves were analyzed by Prism software 5.0.

### miRNAs signature index and survival

We assigned high risk miRNAs expression level as one, or else zero, then scored all miRNAs value in the signature. Therefore, each patient would have a score, named signature index. Input their survival status, survival days and the signature index to plot survival curve using Kaplan-Meier survival method (Log-rank). We set index as high risk and low risk into two group based on the index value. Therefore, all patients were divided into high risk or low risk group according to the miRNAs signature index.

### Chemicals and cell lines

HT126 cells were cultured with DMEM supplemented 10% FBS (Gibico, Thermo Inc.). miRNAs sponge employed pLVX-shRNA2 vector which is purchased from Clonetech Inc. The complete sponge vector was constructed by Genscript Inc. 5-FU was from Selleck Inc.

### Statistics

The levels of miRNAs expression between cancer tissues and non-cancer tissues were analyzed by t-test and p < 0.05 is deemed as significant different. All analysis related with patient survival were testified by Kaplan-Meier survival analysis (Log-rank method). All statistical analyses were performed in Prism 5.0 software.

## Results

### Identification of a 84-miRNAs signature to discriminate colon cancer from non-cancer

Data of 268 COAD patients and 8 controls were downloaded from Harmonized Cancer Datasets (https://gdc-portal.nci.nih.gov/), including miRNAs expression profile, clinical follow-up information, *et al*. The overall information of patients were listed in Table 1. We compared all miRNAs expression level to non-cancer level, and got an 84-miRNAs signature cluster map using Multi-experimental viewer in Hierarchical Clustering method (Supplemental Figure s1). In the clustering analysis, 64 miRNAs were up regulated in cancer tissue, and 20 were down regulated in cancer tissue compared to non-cancer tissue (t test, p < 0.001).

### Validation of 4 miRNAs were associated with patients survival

**Figure 1:**
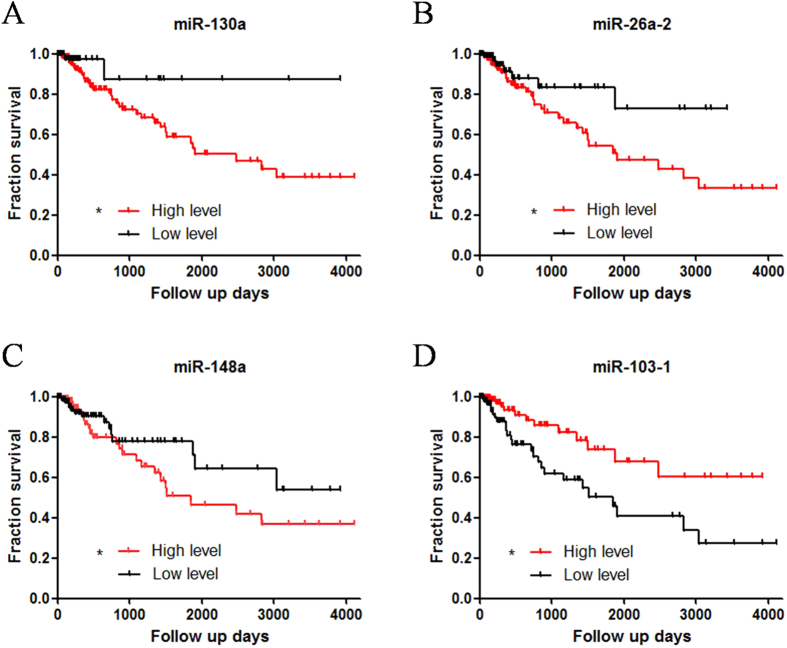
Based on ROC curve cutoff point, miRNAs expression level were divided into two groups: high level expression group and low level expression group. Upon the survival analysis, 4-miRNAs, which was significantly related with patient survival (Log-rank method, *p < 0.05). The high expression level of miR-148a, miR-26a-2 and miR-130a were significantly associated with poor clinical outcome. In miR-130a group, 5-year survival rate was 87.5% in low level group, only 56.3% in high level group (**A**). In miR-26a-2 and miR-148 group, 5-year survival rates were 78.3% and 67.9% in low level group respectively, only 42.8% and 45.2% in high level group (**B**,**C**). Reversely, in miR-103-1, 5-year survival rate was 68.7% in high level group, only 40.5% in low level group (**D**).

Of the above 84 miRNAs, we employed ROC curve method to set the cutoff point to classify 268 patients in two groups: high level group and low level group. If the ROC curve cutoff value was significant, patients were divided into two groups and carried out with Kaplan-Meier survival analysis. Upon the survival analysis, we got 4 miRNAs which were significantly related with patient survival (Fig. 1A, Log-rank method, *p < 0.05). These 4 miRNAs were miR-148a, miR-26a-2, miR-130a and miR-103-1. According to the result, high expression level of miR-148a, miR-26a-2 and miR-130a were regarded as poorer prognostic markers compared to the low level (Fig. 1A–C). As to miR-103-1, contrarily, high expression might be a protection factor in patient survival, while low expression showed a shorter survival rate and time (Fig. 1D). We also performed validation of four miRNAs signature in stage 2 and stage 3 respectively. Data showed miRNAs signature-based risk index was also meaningful in stage 2 and stage 3 patients, indicating the robustness of miRNA signature prognostic biomarker (Supplemental Figure s2).

### Four-miRNAs signature index for colon cancer prognosis

**Figure 2:**
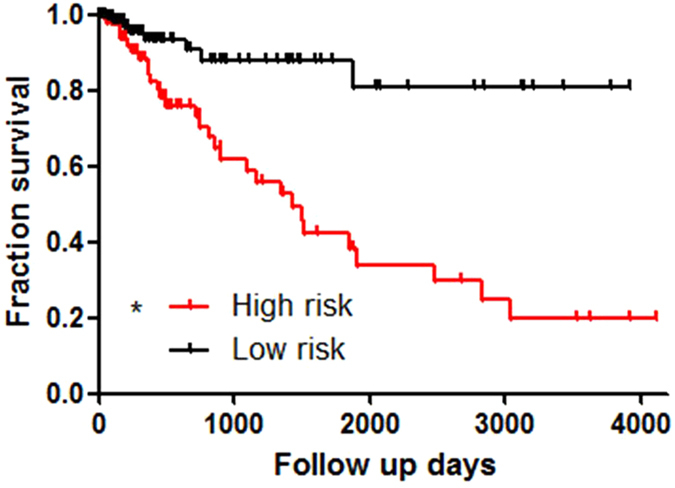
Individual patient was scored according to the four-miRNAs signature. Score 2–4 was ranked as high risk group in survival, and score below 2 as low risk group. The result showed that low risk group had quite a low death rate, the survival rate was 82.7% (**A**). The high risk group had survival rate lower than 20%.

We scored the 4-miRNAs signature by value assignment for each miRNAs. For example, higher expression of miR-148a, miR-26a-2 and miR-130a got one score in each patient, and lower expression of miR-103-1 got one score in each patient. Furthermore, we summated the score for each patient. Accordingly, the highest score would be four, and the lowest would be zero (Supplemental Figure s2). We set index above three as high risk, and those below three as low risk. Therefore, all patients were divided into high risk or low risk group according to the miRNAs index score. We analyzed the two groups by Kaplan-Meier survival analysis in Log-rank method. The result showed that low risk group had quite a low death rate which the survival rate was over 80% (Fig. 2A). The high risk group had survival rate lower than 20%, which was also extremely lower than the overall survival rate in Table 1.

### KEGG signal pathway and GO annotation of 4-miRNAs predicted genes

In order to further explore the four miRNAs signature in biological function and mechanism. We analyzed those potential targets which might be regulated by the four miRNAs through KEGG signal pathway and GO annotation analysis. Firstly, we predicted target genes through online miRNAs prediction software (http://www.microrna.org/microrna/getGeneForm.do), and selected the first 100 predicted targets (Supplemental Figure s3) as input genes for KEGG signal pathway and GO annotation. The results of KEGG pathway analysis were listed in Table 2. The results indicated that cancer related pathways are obviously activated, including glioma, endometrial cancer, thyroid cancer, prostate cancer and leukemia. Several key proteins appealed our interests, such as MDM4, TGFA, CDK19, SHC4 and PTEN. We hypothesized these proteins played vital panel joint in multiple cancers. GO annotation results have three parts: molecular function (Table 3), biological process (Supplemental Figure s4), and cellular component (Supplemental Figure s5). In the molecular function part, we found that many molecular functions were mainly associated with DNA binding and gene transcription. There four miRNAs might be tightly related with gene expression and the cellular and biological function.

### Treatment of miR-130a sponge enhanced anti-cancer drug therapeutic effect in colon cancer cells

**Figure 3:**
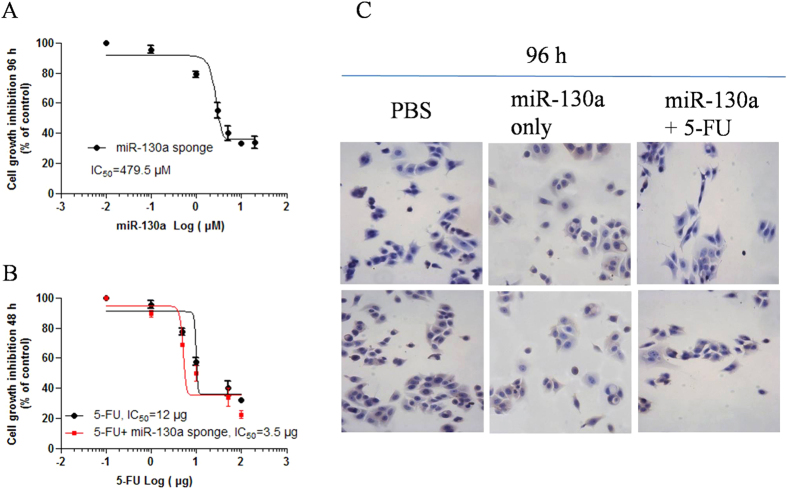
miR-130a sponge vector transfection inhibited HT126 cells growth in a dose dependent manner in 96 h (**A**). The IC50 is 479.5 μM. When combined with anti-cancer drug 5-FU, the miRNA sponge of miR-130a sensitized5-FU anti-cancer effect in 48 h, with the IC50 from 12 μg to 3.5 μg (**B**). In 96 h incubation of transfected miR-130a sponge vector with 5-FU, cell growth was significantly suppressed (**C**).

We used HT126 colon cancer cells in the study to explore the anti-cancer effects of miR-130a sponge. In the study, cell growth assay showed miR-130a sponge inhibited colon cancer cells growth in a dose dependent manner in 96 h (Fig. 3A and B). When it combined with anti-cancer drug 5-FU, the miR-130a sponge sensitized the anti-cancer drug effect of 5-FU to block cancer cell growth in 96 h (Fig. 3C).

## Discussion

miRNAs regulation as a key epigenetic issue, as well as DNA methylation, histone acetylation and methylation, protein modification, would be no doubt becoming important markers in disease diagnosis and prognosis. miRNAs signature have gradually shown its unique and meaningful effects in cancer early diagnosis and survival prognosis. Although the function of miRNAs constituting signature is being increasingly recognized, the mechanism is quite complicated. The survival prognostic miRNAs signature meets many crucial standards. Firstly, the signature must be specific in cancer and non-cancer; secondly, the signature is correlated with patient survival; last, the signature has synergized effect in patient survival prognosis. Several diagnostic and predicted miRNAs signature have revealed by scientists worldwide. It was reported that several miRNAs markers were identified for cancer prediction and prognosis, such as head and neck squamous cell carcinomas^7^, bile duct cancer prediction^8^, and glioma^9^. However, up to date, the miRNAs expression patterns in the survival prognosis of colon cancer have not been investigated systematically. In the present study, we got four miRNAs which were survival associated by using ROC curve method and Kaplan-Meier survival method (p < 0.001) from 467 colon cancer database. We scored the miRNAs signature to form an index by assignment value for each miRNAs. The result showed that low risk group had quite a low death rate, the survival rate of which was over 80%. The high risk group had a survival rate lower than 20%, which was also extremely lower than the overall survival rate. Recently, Gao *et al*. developed 8 cancer hallmark-based gene signature sets which in combination can predict prognostic of recurrence for patient receiving fluorouracil-based chemotherapy of stage 2 colon cancer^12^. In agreement with their study, and enlightening by their idea, we performed validation of four miRNAs signature for stage 2 and stage 3 prognostic analysis, due to the heterogeneous of cancers. To our surprise, not all single miRNA expression level works well for stage 2 and stage 3 patients (data not shown). The combined miRNAs expression level -based index showed association of risk factor with patient survival rate in stage 2 and stage 3 colon cancer patients. The reason might be related with the different potential targets with different weighting effects on cancer hallmark of stage 2 and stage 3, additionally, discrepant regulation on genes expression of each miRNA in different cancer stage.

Wang *et al*. reported that cancer hallmark network framework play important roles in predicting tumor clinical phenotypes^13^. Therefore, we performed GO Terms and KEGG pathway analysis. We also found many predicted genes participated in cancer related pathway and acted oncogene functions. Many genes were involved in the cancer hallmarks^14^, such as cell death (more than 25 genes, p < 0.004, Supplemental Tables s4 and s5), sustained angiogenesis and vessel morphogenesis (more than 12 genes, p < 0.001, Supplemental Table s4), and cellular metabolic processes (more than 47 genes, p = 0.018, Supplemental Table s4). Furthermore, many genes participated in transcription activity (more than 50 genes, Table 3), mRNA processing (more than 5 genes, Table s5), RNA polymerase II transcription factor activity (more than 10 genes, Table 3). These genes were associated with limitless replicative potential, inflammation cytokines release, and immune system^15^,^16^,^17^,^18^. Additionally, we performed KEGG pathway analysis, and found that all pathways were associated with cancer, such as p53 pathway (7 genes, p = 0.002, Table 2), glioma pathway (8 genes, p = 0.001, Table 2), pathway in cancer (14 genes, p = 0.009 Table 2), *et al*. Taken together, the four miRNAs signature potentially regulated cancer related genes, and influenced cancer hallmarks. The vital impacts of these four miRNAs on cancer still need further exploration in the future.

Our observations indicated that the four miRNAs signature may play a critical role in cancer cell growth after anti-cancer drug treatment. To further testify such hypothesis, we constructed miR-130 sponge expression vector and treated cancer cells with anti-cancer drug 5-FU. Results showed miR-130a sponge inhibited HT126 cells growth and sensitized the anti-cancer drug effect of 5-FU to block cancer cell growth. The latest study about gastric cancer reported that miR-130 was an oncogene by directly targeting TGFbetaR2, and as a result, it promotes cancer growth^19^. Egawa H. *et al*. also indicated that the miR-130 family has a crucial role in malignant progression of bladder cancer^20^. The authors suggested the miR-130 family could be a promising therapeutic target for invasive bladder cancer. Some other studies revealed miR-130 also participated in cardiac development and pulmonary hypertension^21^,^22^. So far, rare study focused on miR-130a in colon cancer. In agreement with the previous study of miR-130 as an oncogene, our study further revealed that miR-130a sponge vector could suppressed colon cancer cell growth. The miR-130a sponge also sensitized 5-FU drug anti-cancer effect. The influence of miR-130a expression on regulation of cell growth and anti-cancer drug efficacy might be through targeting some vital signal pathway members. In the miR-130a targets prediction, many potential genes play important roles in cell growth (MDM4, KLF7, ACVR1, IRF1, DYNLL2 *et al*.), transcription activity (MDM4, MYBL1, ACVR1, MAF *et al*.), mRNA processing (KLF7, CPEB1, IRF1, RPS6KA5 *et al*.), protein kinase activity (PAN3, TGFBR2, CDK19, RPS6KA5 *et al*.) and metabolic process (PAN3, CPEB1, RPS6KA5, SBF2 *et al*.). Therefore, we hypothesized that miR-130a might act as a anti-cancer target, as well as a prognostic biomarker for colon cancer.

Our results demonstrated that the four-miRNAs signature is a potential marker for colon cancer patient survival prognosis. The suppressing effect of colon cancer cell growth of miR-130a sponge vector supplies a brand new insight into the therapeutic strategy against colon cancer, especially for those patients with poor predicted survival rate.

## Additional Information

**How to cite this article**: Xu, J. *et al*. Four microRNAs Signature for Survival Prognosis in Colon Cancer using TCGA Data. *Sci. Rep.* **6**, 38306; doi: 10.1038/srep38306 (2016).

**Publisher's note:** Springer Nature remains neutral with regard to jurisdictional claims in published maps and institutional affiliations.

## Supplementary Material

Supplementary Data

## Figures and Tables

**Table 1:** Summary of patient cohort information.

Characteristic	Cohort (n = 268)	
	No.	%
Sex		
Male	146	54.48
Female	122	45.52
Age, years		
Median	61	
Range	17–85	
Height, cm		
Median	170	
Range	80.3–193	
Weight, kg		
Median	80	
Range	34–175.3	
Histological classification		
Colon Mucinous Adenocarcinoma	40	14.93
Colon Adenocarcinoma	228	85.07
CEA+ level pretreatment		
Median	3.4	
Range	0.2–1286	
Pathologic stage		
Stage I	39	14.55
Stage II	106	39.55
Stage III	81	30.22
Stage IV	34	12.69
Not known	8	2.99
Tissue invasion		
Vascular invasion	51/237	21.52
Lymphovascular invasion	67/239	28.03
Perineural invasion	43/165	26.06
Follow-up, days		
Median	182	
Range	0–4122	
Mean	534.3	
SD	869.4	
Survival rate		
1 year	71	82.60
3 year	44	61.11
5 year	24	35.82

**Table 2:** KEGG pathway analysis of predicted targets from the four miRNAs.

Pathway	Count	Genes	p value
hsa05214:Glioma	8	NRAS, SOS1, SOS2, TGFA, IGF1, PTEN, CALM2, SHC4	0.001
hsa04115:p53 signaling pathway	7	BID, ZMAT3, IGF1, PMAIP1, MDM4, GADD45A, PTEN	0.002
hsa05200:Pathways in cancer	14	BID, NRAS, CCDC6, RET, SOS1, SOS2, PPARG, TGFBR2, MITF, SKP2, TGFA, IGF1, AXIN2, PTEN	0.009
hsa04722:Neurotrophin signaling pathway	8	RPS6KA5, NRAS, ZNF274, SOS1, SOS2, PRKCD, CALM2, SHC4	0.009
hsa04910:Insulin signaling pathway	8	PRKAG3, NRAS, TSC1, SOS1, SOS2, RHOQ, CALM2, SHC4	0.014
hsa05213:Endometrial cancer	5	NRAS, SOS1, SOS2, AXIN2, PTEN	0.017
hsa05216:Thyroid cancer	4	NRAS, CCDC6, RET, PPARG	0.017
hsa04012:ErbB signaling pathway	6	NRAS, EREG, SOS1, SOS2, TGFA, SHC4	0.025
hsa05215:Prostate cancer	6	NRAS, SOS1, SOS2, TGFA, IGF1, PTEN	0.027
hsa05220:Chronic myeloid leukemia	5	NRAS, SOS1, SOS2, TGFBR2, SHC4	0.054

**Table 3:** GO annotation: cellular function of predicted targets from the four miRNAs.

Term	Count	p Value	Genes
GO:0003700 transcription factor activity	34	0.001	SLC2A4RG, ZNF274, TSHZ1, MITF, PPARG, CBFB, ATF2, HOXA5, FOSL1, MAF, KLF5, KLF6, KLF7, CREBZF, MAFB, BARHL2, OTX2, ZHX1, ESR1, SMAD1, MXD1, HMGA1, TRERF1, ZNF3, HOXB1, TAF13, ZNF217, MTF1, MEOX2, IRF1, MNX1, ST18, NFIA, KLF3
GO:0030528 transcription regulator activity	47	0.001	ZNF274, SLC2A4RG, TSHZ1, MITF, PPARG, EZH2, MYBL1, CBFB, ATF2, EPC1, NPAS3, HOXA5, MED26, HBP1, FOSL1, MAF, KLF5, KLF6, KLF7, CREBZF, MAFB, ZHX1, BARHL2, OTX2, ESR1, PPP1R10, SMAD1, MXD1, HMGA1, TRERF1, ZNF3, NRIP1, CDKN1C, HOXB1, TAF13, ZNF217, MTF1, CD80, MEOX2, BTG1, UBC, MNX1, IRF1, ST18, NFIA, TOB1, KLF3
GO:0016563 transcription activator activity	17	0.006	KLF6, KLF7, MITF, PPARG, SMAD1, MYBL1, TRERF1, HMGA1, CBFB, NRIP1, ATF2, CDKN1C, EPC1, CD80, MTF1, MED26, FOSL1
GO:0046965 retinoid X receptor binding	3	0.01	PPARG, HMGA1, NRIP1
GO:0017124 SH3 domain binding	7	0.011	CCDC6, SOS1, GJA1, QKI, ARHGAP17, NCKIPSD, ADAM9
GO:0019904 protein domain specific binding	14	0.013	RHOQ, GJA1, ARHGAP17, PTEN, ATP2B2, CCDC6, HOXB1, SOS1, QKI, NCKIPSD, CALM2, PMEPA1, ADAM9, SHC4
GO:0004672 protein kinase activity	21	0.014	CDK19, PRKAG3, RET, TWF1, CDK5R1, PAN3, FGFRL1, TGFBR2, CDK8, NEK10, STRADB, PRKCE, PRKCD, EPHA3, RPS6KA5, ULK2, DYRK1A, TGFA, STK39, KALRN, ACVR1
GO:0003924~GTPase activity	10	0.022	NRAS, RAP2C, MRAS, EIF5, RAB34, RHOQ, ARL8B, RHOU, GBP3, RRAGC
GO:0017076 purine nucleotide binding	49	0.036	CDK19, UBE2G1, EIF5, RHOQ, INO80, KCNJ10, RHOU, ACTR3, KIF2B, ATP2B2, STK39, DUS1L, CDC6, RET, RAP2C, PAN3, CDK8, NEK10, CCT6A, PRKCE, PRKCD, UBE2W, ARL8B, GBP3, KALRN, ACVR1, HS3ST5, MRAS, RRAGD, TK2, RRAGC, PALM3, ACSL3, HELLS, TGFBR2, STRADB, ABCB7, EPHA3, ATP13A4, DDX6, RPS6KA5, NRAS, CKMT1A, ULK2, DYRK1A, RAB34, IPPK, CLCN6, DDX52
GO:0004699calcium-independent protein kinase C activity	2	0.038	PRKCE, PRKCD
GO:0032553 ribonucleotide binding	47	0.039	CDK19, MRAS, UBE2G1, EIF5, INO80, RHOQ, KCNJ10, RRAGD, RHOU, TK2, RRAGC, ACTR3, KIF2B, ATP2B2, PALM3, STK39, ACSL3, HELLS, CDC6, RET, PAN3, RAP2C, TGFBR2, CDK8, NEK10, CCT6A, STRADB, PRKCE, ABCB7, PRKCD, EPHA3, DDX6, ATP13A4, RPS6KA5, NRAS, CKMT1A, ULK2, DYRK1A, RAB34, UBE2W, ARL8B, CLCN6, IPPK, GBP3, DDX52, KALRN, ACVR1
GO:0032555 purine ribonucleotide binding	47	0.039	CDK19, MRAS, UBE2G1, EIF5, INO80, RHOQ, KCNJ10, RRAGD, RHOU, TK2, RRAGC, ACTR3, KIF2B, ATP2B2, PALM3, STK39, ACSL3, HELLS, CDC6, RET, PAN3, RAP2C, TGFBR2, CDK8, NEK10, CCT6A, STRADB, PRKCE, ABCB7, PRKCD, EPHA3, DDX6, ATP13A4, RPS6KA5, NRAS, CKMT1A, ULK2, DYRK1A, RAB34, UBE2W, ARL8B, CLCN6, IPPK, GBP3, DDX52, KALRN, ACVR1
GO:0003723RNA binding	22	0.04	EIF4E3, AGFG1, CPEB2, CPEB3, ZMAT3, RPUSD1, PPP1R10, BICC1, CPEB1, ELAVL4, SRP19, SNURF, DDX6, FXR1, PHAX, TROVE2, DCP2, RPL34, QKI, STRBP, TNRC6A, DDX52
GO:0042974 retinoic acid receptor binding	3	0.042	PPARG, HMGA1, NRIP1
GO:0003702RNA polymerase II transcription factor activity	10	0.049	KLF5, MAF, TAF13, MED26, MITF, MNX1, SMAD1, TRERF1, CBFB, ATF2
GO:0043565 sequence-specific DNA binding	19	0.05	MAF, TSHZ1, CREBZF, MAFB, ZHX1, PPARG, OTX2, BARHL2, MITF, ESR1, HMGA2, HMGA1, ATF2, HOXB1, MEOX2, HOXA5, IRF1, MNX1, FOSL1
