# Supplementary material for: Four microRNAs Signature for Survival Prognosis in Colon Cancer using TCGA Data
Source: Sci Rep. 2016 Dec 15;6:38306. doi: 10.1038/srep38306 (PMC5156922; doi:10.1038/srep38306)
Supplement: Supplementary Data [file srep38306-s1.pdf]

Four microRNAs Signature for Survival Prognosis in Colon Cancer using TCGA Data

Jian Xu, Jian Zhao, Rui Zhang*

Department of Colorectal Surgery, Cancer Hospital of China Medical University, Liaoning Cancer Hospital & Institute

Supplemental figure s1

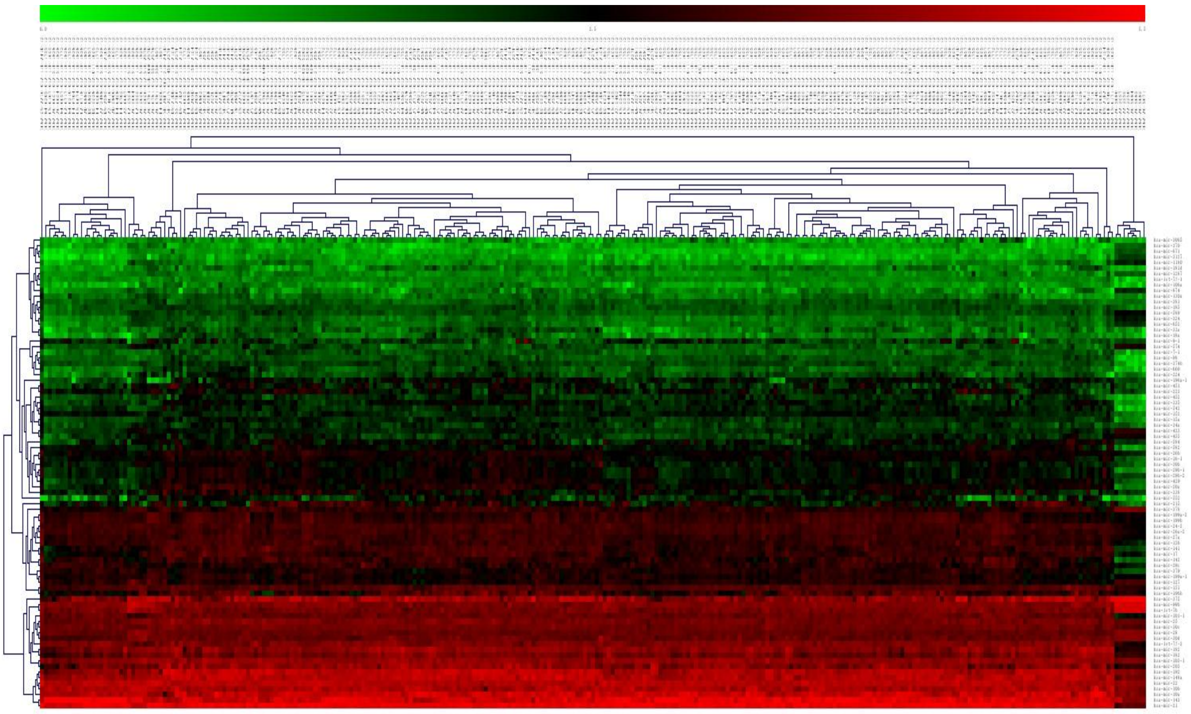

Supplemental figure s1. Hierarchical clustering of 84-miRNAs to classify cancer samples and non-cancer samples.

Supplemental figure s2

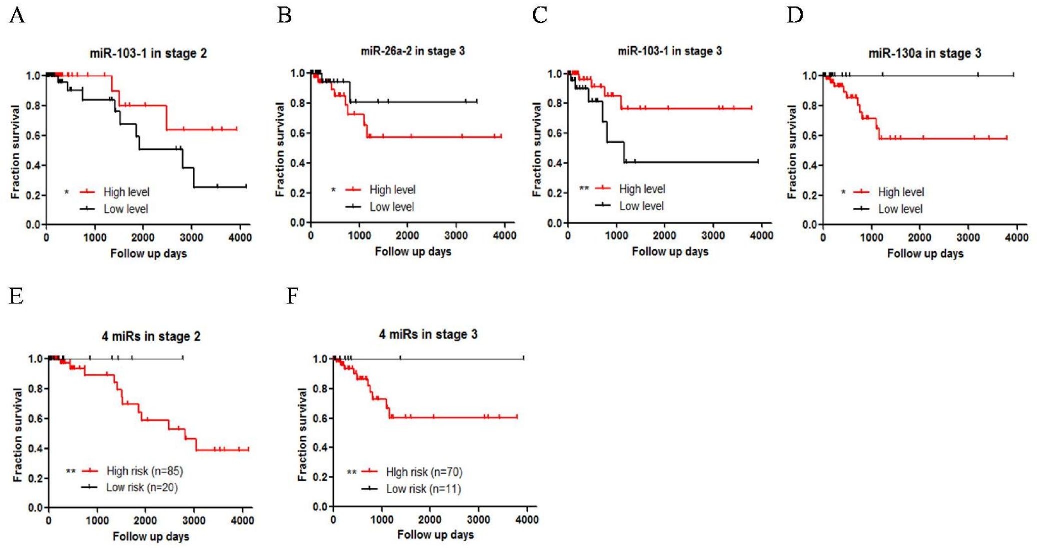

Supplemental figure s2. Four miRNAs signature in colon cancer stage 2 and stage 3. In the Kaplan-Meier survival analysis of four miRNAs one by one, results showed that only miR-103-1 was significant in stage 2 (A), while miR-26a-2, miR-103-1 and miR-130a were significant in stage 3 of patient survival (B, C and D). In the risk factor ranking aspect, score 2-4 was ranked as high risk, and score below 2 as low risk. Data showed the four miRNAs signature was significant both in stage 2 and stage 3 (E and F, **p<0.01).

Supplemental table s1. Summary of 84 microRNAs expression level

microRNA	Mean of Tumor (log, n=268)	Mean of Nontumor (log, n=8)	Expression level in colon cancer
hsa-mir-21	5.31±0.02	3.57±0.03	Up
hsa-mir-101-1	3.75±0.03	2.31±0.02	Up
hsa-mir-379	2.89±0.03	1.7±0.01	Up
hsa-mir-98	1.71±0.03	0.5±0.03	Up
hsa-mir-542	2.18±0.03	0.6±0.04	Up
hsa-mir-335	2.26±0.04	0.96±0.03	Up
hsa-mir-452	2.06±0.04	0.68±0.04	Up
hsa-mir-141	3.15±0.04	1.87±0.01	Up
hsa-mir-182	4.16±0.04	2.87±0.02	Up
hsa-mir-142	3.07±0.05	1.49±0.03	Up
hsa-let-7f-2	4.01±0.03	2.84±0.03	Up
hsa-mir-29b-1	2.48±0.04	1.12±0.03	Up
hsa-mir-582	2.43±0.04	1.05±0.04	Up
hsa-mir-20a	2.69±0.05	1.29±0.03	Up
hsa-mir-203	4.09±0.05	3.06±0.03	Up
hsa-mir-126	3.16±0.03	2.27±0.03	Up
hsa-mir-552	2.12±0.08	0.81±0.04	Up
hsa-mir-429	2.53±0.05	1.6±0.03	Up
hsa-mir-152	2.26±0.03	1.3±0.02	Up
hsa-mir-374b	1.87±0.03	0.7±0.04	Up
hsa-mir-15a	2.09±0.03	1.11±0.04	Up
hsa-mir-660	1.67±0.04	0.61±0.04	Up
hsa-mir-183	3.85±0.04	3.18±0.02	Up
hsa-mir-10a	4.82±0.04	4.02±0.03	Up
hsa-mir-16-1	2.61±0.03	1.64±0.03	Up
hsa-mir-17	3.08±0.04	2.28±0.03	Up
hsa-mir-29b-2	2.49±0.04	1.91±0.01	Up
hsa-mir-148a	4.63±0.04	3.79±0.03	Up
hsa-mir-29c	2.99±0.04	2.3±0.03	Up
hsa-mir-199b	3.17±0.03	2.62±0.02	Up
hsa-mir-30b	2.44±0.04	1.64±0.04	Up
hsa-mir-7-1	1.65±0.04	0.88±0.05	Up
hsa-mir-181d	1.28±0.05	0.75±0.02	Up
hsa-mir-196a-1	2.24±0.07	1.78±0.02	Up
hsa-mir-199a-1	2.84±0.03	2.52±0.01	Up
hsa-mir-192	4.6±0.05	4.06±0.02	Up
hsa-mir-223	2.41±0.06	1.8±0.03	Up
hsa-mir-215	2.64±0.08	1.51±0.06	Up
hsa-mir-151	3.45±0.03	2.96±0.02	Up
hsa-mir-451	2.28±0.06	1.7±0.03	Up
hsa-mir-199a-2	3.07±0.03	2.69±0.01	Up
hsa-mir-143	5.14±0.05	4.24±0.05	Up
hsa-mir-26b	2.74±0.03	2.22±0.02	Up
hsa-mir-33a	1.49±0.06	0.95±0.05	Up
hsa-mir-10b	4.52±0.03	4.01±0.03	Up
hsa-mir-338	2.51±0.06	1.99±0.03	Up
hsa-mir-224	1.8±0.05	1.35±0.04	Up
hsa-mir-27a	3.16±0.03	2.63±0.02	Up
hsa-mir-25	3.94±0.03	3.68±0.01	Up
hsa-mir-24-2	3.29±0.02	2.7±0.02	Up
hsa-mir-196b	3.16±0.07	2.87±0.04	Up
hsa-mir-106a	1.06±0.04	0.58±0.04	Up
hsa-mir-22	4.67±0.02	4.22±0.02	Up
hsa-mir-30e	3.86±0.02	3.47±0.02	Up
hsa-mir-3065	1.01±0.05	0.76±0.02	Up
hsa-mir-34a	2.02±0.04	1.51±0.04	Up
hsa-mir-26a-2	3.23±0.02	2.78±0.03	Up
hsa-mir-381	1.63±0.03	1.43±0.01	Up
hsa-mir-130a	1.41±0.04	1.2±0.03	Up
hsa-mir-18a	1.35±0.06	0.87±0.05	Up
hsa-let-7f-1	1.23±0.02	0.59±0.04	Up
hsa-mir-9-1	2.01±0.06	1.85±0.01	Up
hsa-mir-103-1	4.22±0.03	3.96±0.02	Up
hsa-mir-185	1.77±0.03	1.38±0.03	Up
hsa-mir-584	2.38±0.06	2±0.02	Down
hsa-let-7b	3.91±0.03	5.03±0.01	Down

hsa-mir-28	3.71±0.02	4.12±0.01	Down
hsa-mir-30d	3.7±0.03	4.09±0.01	Down
hsa-mir-423	2.14±0.03	3.06±0.01	Down
hsa-mir-375	4.29±0.06	5.4±0.01	Down
hsa-mir-378	3.02±0.04	3.94±0.01	Down
hsa-mir-589	1.72±0.03	2.49±0.01	Down
hsa-mir-455	2.1±0.04	2.73±0.01	Down
hsa-mir-127	2.87±0.04	3.37±0.01	Down
hsa-mir-574	1.73±0.04	2.87±0.01	Down
hsa-mir-324	1.45±0.03	2.29±0.01	Down
hsa-mir-1180	1.1±0.04	2.21±0.01	Down
hsa-mir-874	1.24±0.05	2.11±0.01	Down
hsa-mir-3127	0.82±0.04	1.88±0.02	Down
hsa-mir-671	0.81±0.03	1.67±0.01	Down
hsa-mir-370	0.88±0.04	1.56±0.01	Down
hsa-mir-1287	1.18±0.03	1.62±0.01	Down
hsa-mir-652	1.46±0.04	2.1±0.01	Down
hsa-mir-99b	4.05±0.03	5.08±0.01	Down

Supplemental table s2. Four miRNAs signature for survival analysis

Patients	Status(dead =1, alive=0)	Follow up days	miR-148a	miR-26a-2	miR-130a	miR-103-1	miRNA Index
TCGA-G4-6626	1	1422	1	1	1	1	4
TCGA-G4-6294	1	858	1	1	1	1	4
TCGA-AZ-6608	1	59	1	1	1	1	4
TCGA-AZ-6606	1	357	1	1	1	1	4
TCGA-AZ-6603	1	899	1	1	1	1	4
TCGA-AZ-6600	1	368	1	1	1	1	4
TCGA-AZ-6599	1	206	1	1	1	1	4
TCGA-DM-A1D4	1	2821	1	1	1	1	4
TCGA-DM-A28G	1	1849	1	1	1	1	4
TCGA-DM-A1D6	1	1518	1	1	1	1	4
TCGA-DM-A0XF	1	1162	1	1	1	1	4
TCGA-DM-A288	1	427	1	1	1	1	4
TCGA-DM-A1D8	1	383	1	1	1	1	4
TCGA-DM-A1D7	1	0	1	1	1	1	4
TCGA-DM-A282	0	4122	1	1	1	1	4
TCGA-G4-6625	0	2673	1	1	1	1	4
TCGA-G4-6306	0	1359	1	1	1	1	4
TCGA-A6-2672	0	503	1	1	1	1	4
TCGA-G4-6295	0	254	1	1	1	1	4
TCGA-A6-6138	0	182	1	1	1	1	4
TCGA-A6-6649	0	147	1	1	1	1	4
TCGA-CM-6680	0	91	1	1	1	1	4
TCGA-NH-A6GC	0	40	1	1	1	1	4
TCGA-AA-3496	0	31	1	1	1	1	4
TCGA-F4-6806	0	20	1	1	1	1	4
TCGA-F4-6808	0	14	1	1	1	1	4
TCGA-4N-A93T	0	8	1	1	1	1	4
TCGA-CM-5862	1	153	1	1	1	0	3
TCGA-AZ-6598	1	1503	1	1	1	0	3
TCGA-A6-A565	1	494	1	1	1	0	3
TCGA-5M-AAT6	1	290	1	1	1	0	3
TCGA-DM-A28C	1	2475	1	1	1	0	3
TCGA-DM-A1DB	1	1348	1	1	1	0	3
TCGA-DM-A28F	1	1094	1	1	1	0	3
TCGA-DM-A1D9	0	3920	1	1	1	0	3
TCGA-DM-A1D0	0	3625	1	1	1	0	3
TCGA-5M-AATE	0	1200	1	1	1	0	3
TCGA-G4-6317	0	894	1	1	1	0	3
TCGA-G4-6320	0	678	1	1	1	0	3
TCGA-CK-4947	0	534	1	1	1	0	3
TCGA-D5-5537	0	456	1	1	1	0	3
TCGA-AU-3779	0	441	1	1	1	0	3
TCGA-QL-A97D	0	295	1	1	1	0	3
TCGA-D5-6529	0	260	1	1	1	0	3
TCGA-CM-6172	0	212	1	1	1	0	3
TCGA-AY-A8YK	0	209	1	1	1	0	3
TCGA-G4-6323	0	201	1	1	1	0	3
TCGA-A6-6137	0	195	1	1	1	0	3
TCGA-A6-6650	0	173	1	1	1	0	3
TCGA-NH-A6GB	0	170	1	1	1	0	3
TCGA-NH-A8F7	0	158	1	1	1	0	3
TCGA-A6-6141	0	130	1	1	1	0	3
TCGA-AD-6888	0	128	1	1	1	0	3

TCGA-A6-6140	0	125	1	1	1	0	3
TCGA-D5-6532	0	107	1	1	1	0	3
TCGA-D5-6535	0	10	1	1	1	0	3
TCGA-T9-A92H	0	0	1	1	1	0	3
TCGA-F4-6461	0	0	1	1	1	0	3
TCGA-AA-3511	0	0	1	1	1	0	3
TCGA-G4-6298	1	715	0	1	1	1	3
TCGA-AZ-6605	1	159	0	1	1	1	3
TCGA-AZ-6601	1	3042	0	1	1	1	3
TCGA-AZ-5403	1	1910	0	1	1	1	3
TCGA-AZ-4616	1	156	0	1	1	1	3
TCGA-AZ-4323	1	43	0	1	1	1	3
TCGA-A6-4105	1	442	1	0	1	1	3
TCGA-DM-A28A	1	805	1	0	1	1	3
TCGA-DM-A0XD	1	743	0	1	1	1	3
TCGA-DM-A280	1	236	0	1	1	1	3
TCGA-DM-A28E	0	3530	0	1	1	1	3
TCGA-G4-6303	0	1882	0	1	1	1	3
TCGA-G4-6628	0	1606	0	1	1	1	3
TCGA-G4-6310	0	1205	0	1	1	1	3
TCGA-G4-6586	0	741	0	1	1	1	3
TCGA-CM-4751	0	607	0	1	1	1	3
TCGA-G4-6321	0	572	0	1	1	1	3
TCGA-A6-2675	0	515	0	1	1	1	3
TCGA-CM-6164	0	487	0	1	1	1	3
TCGA-CM-5861	0	335	0	1	1	1	3
TCGA-A6-5661	0	306	0	1	1	1	3
TCGA-CM-5863	0	304	0	1	1	1	3
TCGA-CM-5860	0	304	0	1	1	1	3
TCGA-A6-5665	0	286	0	1	1	1	3
TCGA-D5-6530	0	271	1	1	0	1	3
TCGA-A6-6648	0	253	1	1	0	1	3
TCGA-NH-A6GA	0	182	1	0	1	1	3
TCGA-A6-6654	0	178	0	1	1	1	3
TCGA-A6-6782	0	164	0	1	1	1	3
TCGA-NH-A8F7	0	158	1	0	1	1	3
TCGA-3L-AA1B	0	154	1	0	1	1	3
TCGA-CM-6677	0	153	0	1	1	1	3
TCGA-CM-6676	0	153	0	1	1	1	3
TCGA-CM-6674	0	150	0	1	1	1	3
TCGA-D5-6929	0	146	1	0	1	1	3
TCGA-CM-6679	0	122	0	1	1	1	3
TCGA-CM-6678	0	122	0	1	1	1	3
TCGA-D5-6920	0	112	1	0	1	1	3
TCGA-D5-6924	0	89	1	0	1	1	3
TCGA-CK-6747	0	89	1	0	1	1	3
TCGA-F4-6703	0	56	0	1	1	1	3
TCGA-F4-6704	0	47	0	1	1	1	3
TCGA-F4-6855	0	42	0	1	1	1	3
TCGA-F4-6809	0	39	0	1	1	1	3
TCGA-AD-6901	0	31	0	1	1	1	3
TCGA-AA-3489	0	31	0	1	1	1	3
TCGA-D5-5539	0	30	0	1	1	1	3
TCGA-D5-6932	0	19	0	1	1	1	3
TCGA-F4-6854	0	16	1	0	1	1	3
TCGA-AD-6963	0	3	0	1	1	1	3
TCGA-AD-6548	0	3	0	1	1	1	3
TCGA-AD-5900	0	2	0	1	1	1	3
TCGA-F4-6570	0	0	0	1	1	1	3
TCGA-F4-6569	0	0	0	1	1	1	3
TCGA-F4-6460	0	0	1	0	1	1	3
TCGA-F4-6459	0	0	0	1	1	1	3
TCGA-A6-A566	1	758	0	1	1	0	2
TCGA-DM-A1DA	1	228	1	0	1	0	2
TCGA-DM-A1HB	0	3786	0	1	1	0	2
TCGA-DM-A0X9	0	3429	1	0	1	0	2
TCGA-DM-A28H	0	3423	1	0	1	0	2
TCGA-CK-4948	0	3116	0	1	1	0	2
TCGA-DM-A28K	0	2835	1	0	1	0	2
TCGA-G4-6299	0	2077	0	1	1	0	2
TCGA-WS-AB45	0	2038	1	0	1	0	2
TCGA-A6-A56B	0	1595	1	0	1	0	2
TCGA-G4-6307	0	1485	0	1	1	0	2
TCGA-CK-5912	0	1466	1	1	0	0	2
TCGA-CK-4950	0	1227	1	1	0	0	2
TCGA-G4-6311	0	1112	0	1	1	0	2
TCGA-G4-6315	0	937	0	1	1	0	2
TCGA-RU-A8FL	0	921	1	0	1	0	2
TCGA-G4-6317	0	894	0	1	1	0	2
TCGA-QG-A5YV	0	821	1	0	1	0	2
TCGA-G4-6588	0	644	0	1	1	0	2

TCGA-A6-5656	0	462	0	1	1	0	2
TCGA-A6-5656	0	462	0	1	1	0	2
TCGA-QG-A5Z2	0	449	1	0	1	0	2
TCGA-QG-A5YW	0	399	1	0	1	0	2
TCGA-A6-5660	0	342	0	1	1	0	2
TCGA-CM-5349	0	335	0	1	1	0	2
TCGA-CM-5868	0	304	0	1	1	0	2
TCGA-D5-6536	0	287	1	1	0	0	2
TCGA-CM-6169	0	243	0	1	1	0	2
TCGA-CM-6170	0	242	0	1	1	0	2
TCGA-D5-6534	0	241	0	1	1	0	2
TCGA-AY-A71X	0	235	1	0	1	0	2
TCGA-AY-5543	0	230	1	1	0	0	2
TCGA-CM-6165	0	212	0	1	1	0	2
TCGA-CM-6162	0	212	0	1	1	0	2
TCGA-CM-6171	0	182	1	1	0	0	2
TCGA-A6-6650	0	173	1	0	1	0	2
TCGA-A6-6650	0	173	1	0	1	0	2
TCGA-AY-A69D	0	168	1	0	1	0	2
TCGA-NH-A8F8	0	167	0	1	1	0	2
TCGA-4T-AA8H	0	160	1	0	1	0	2
TCGA-AY-A54L	0	158	1	0	1	0	2
TCGA-A6-6781	0	158	1	0	1	0	2
TCGA-A6-6781	0	158	0	1	1	0	2
TCGA-A6-6781	0	158	0	1	1	0	2
TCGA-NH-A50V	0	154	1	0	1	0	2
TCGA-D5-6539	0	145	1	1	0	0	2
TCGA-A6-6652	0	135	1	0	1	0	2
TCGA-A6-6142	0	134	0	1	1	0	2
TCGA-CM-6167	0	122	0	1	1	0	2
TCGA-A6-A5ZU	0	117	1	0	1	0	2
TCGA-D5-6541	0	108	0	1	1	0	2
TCGA-CM-6163	0	90	0	1	1	0	2
TCGA-A6-6780	0	87	0	1	1	0	2
TCGA-NH-A5IV	0	85	0	1	1	0	2
TCGA-AD-6899	0	48	1	0	1	0	2
TCGA-AA-3660	0	31	1	1	0	0	2
TCGA-AA-3697	0	30	1	1	0	0	2
TCGA-AD-6964	0	23	0	1	1	0	2
TCGA-D5-6928	0	20	0	1	1	0	2
TCGA-AD-A5EK	0	13	1	0	1	0	2
TCGA-CA-5797	0	8	0	1	1	0	2
TCGA-AY-6196	0	6	0	1	1	0	2
TCGA-CA-5255	0	2	0	1	1	0	2
TCGA-F4-6463	0	0	0	1	1	0	2
TCGA-AA-3712	0	0	0	1	1	0	2
TCGA-AA-3662	0	0	1	1	0	0	2
TCGA-CK-6746	0	-2	1	1	0	0	2
TCGA-AZ-6607	1	97	0	1	0	1	2
TCGA-DM-A285	1	186	0	0	1	1	2
TCGA-DM-A1HA	0	3924	0	1	0	1	2
TCGA-CK-6751	0	3136	0	0	1	1	2
TCGA-DM-A28M	0	2775	0	0	1	1	2
TCGA-G4-6627	0	1434	0	1	0	1	2
TCGA-G4-6309	0	1386	0	0	1	1	2
TCGA-G4-6302	0	1310	0	0	1	1	2
TCGA-G4-6314	0	1031	0	0	1	1	2
TCGA-CM-6166	0	424	0	0	1	1	2
TCGA-A6-5657	0	367	0	0	1	1	2
TCGA-A6-5664	0	362	0	0	1	1	2
TCGA-CM-5344	0	304	0	0	1	1	2
TCGA-A6-6651	0	249	0	0	1	1	2
TCGA-D5-6922	0	134	0	0	1	1	2
TCGA-D5-6927	0	67	0	0	1	1	2
TCGA-CK-6748	0	61	0	0	1	1	2
TCGA-D5-6930	0	46	0	0	1	1	2
TCGA-D5-6898	0	45	0	0	1	1	2
TCGA-D5-6931	0	43	1	0	0	1	2
TCGA-D5-6923	0	43	0	0	1	1	2
TCGA-D5-7000	0	41	0	0	1	1	2
TCGA-D5-6533	0	40	0	1	0	1	2
TCGA-AD-6890	0	24	0	0	1	1	2
TCGA-AD-6895	0	22	0	0	1	1	2
TCGA-F4-6807	0	16	0	0	1	1	2
TCGA-F4-6805	0	16	0	0	1	1	2
TCGA-AD-6965	0	6	0	0	1	1	2
TCGA-CA-5254	0	5	0	0	1	1	2
TCGA-CA-6718	0	3	0	0	1	1	2
TCGA-NH-A50U	1	334	0	0	1	0	1
TCGA-CK-5916	1	643	0	1	0	0	1
TCGA-A6-A567	1	1881	0	0	1	0	1

TCGA-5M-AAT4	1	49	0	0	1	0	1
TCGA-G4-6293	0	3203	1	0	0	0	1
TCGA-G4-6297	0	2289	0	1	0	0	1
TCGA-G4-6304	0	1631	0	0	1	0	1
TCGA-AZ-5407	0	1395	1	0	0	0	1
TCGA-CK-5914	0	669	0	0	1	0	1
TCGA-G4-6322	0	547	1	0	0	0	1
TCGA-QG-A5YX	0	526	0	0	1	0	1
TCGA-CK-4952	0	475	1	0	0	0	1
TCGA-A6-5656	0	462	0	0	1	0	1
TCGA-CM-5348	0	393	0	1	0	0	1
TCGA-A6-5667	0	320	0	0	1	0	1
TCGA-A6-5662	0	297	0	1	0	0	1
TCGA-A6-5659	0	286	0	0	1	0	1
TCGA-D5-6538	0	246	1	0	0	0	1
TCGA-A6-5666	0	244	1	0	0	0	1
TCGA-D5-5538	0	204	0	1	0	0	1
TCGA-QG-A5Z1	0	202	0	0	1	0	1
TCGA-D5-5541	0	202	0	1	0	0	1
TCGA-CM-4743	0	184	0	1	0	0	1
TCGA-CM-6168	0	181	0	0	1	0	1
TCGA-AY-6386	0	169	1	0	0	0	1
TCGA-NH-A50T	0	125	0	0	1	0	1
TCGA-D5-6926	0	95	0	0	1	0	1
TCGA-A6-6653	0	91	0	0	1	0	1
TCGA-A6-6780	0	87	1	0	0	0	1
TCGA-CM-5864	0	61	1	0	0	0	1
TCGA-AA-3663	0	31	1	0	0	0	1
TCGA-AA-3713	0	30	0	1	0	0	1
TCGA-F4-6856	0	20	0	0	1	0	1
TCGA-CA-5796	0	12	1	0	0	0	1
TCGA-CA-6715	0	2	1	0	0	0	1
TCGA-CK-5915	0	0	1	0	0	0	1
TCGA-AD-A5EJ	0	0	0	0	1	0	1
TCGA-AA-3655	0	0	1	0	0	0	1
TCGA-A6-5665	0	286	0	0	0	1	1
TCGA-CM-6675	0	153	0	0	0	1	1
TCGA-CA-6719	0	41	0	0	0	1	1
TCGA-CA-6717	0	10	0	0	0	1	1
TCGA-CA-6716	0	8	0	0	0	1	1
TCGA-AD-6889	0	1720	0	0	0	0	0
TCGA-CK-5913	0	853	0	0	0	0	0
TCGA-A6-5661	0	306	0	0	0	0	0
TCGA-CM-6161	0	212	0	0	0	0	0
TCGA-AU-6004	0	203	0	0	0	0	0
TCGA-D5-5540	0	198	0	0	0	0	0
TCGA-D5-6540	0	186	0	0	0	0	0
TCGA-D5-6537	0	137	0	0	0	0	0
TCGA-AY-6197	0	113	0	0	0	0	0
TCGA-A6-6780	0	87	0	0	0	0	0
TCGA-AM-5821	0	28	0	0	0	0	0
TCGA-D5-6531	0	17	0	0	0	0	0
TCGA-AM-5820	0	14	0	0	0	0	0

Supplemental table s3. Gene targets of four miRNAs

miR-148a	miR-26a-2	miR-130a	miR-103-1
ATP6AP2	POLR3G	SLAIN1	FGFRL1
MEOX2	CHORDC1	MDM4	RNF38
GLRX5	THAP2	KDM2A	PPP6R2
C5orf30	STRADB	KLF7	TRIAP1
SNN	ARPP19	PAN3	MED26
GADD45A	MTX2	ENPP5	GJE1
ARL8B	ZDHHC6	FBXO28	NPAS3
NPTN	ART3	MYBL1	ANO3
NPEPL1	MAB21L1	ACVR1	PRKAG3
MITF	BID	CPEB1	ARIH2
CLCN6	SRP19	CHST1	NEK10
CDK5R1	PALM3	RTCA	ARMC1
TMEM54	FAM98A	SYBU	FAM103A1
C18orf25	SERP1	IRF1	AGFG1
SOS2	CREBZF	RPS6KA5	TFRC
HLA-DQB2	GMDS	MAF	NEIL1
S1PR1	HMGA1	DYNLL2	CACNA2D1
QKI	EZH2	SBF2	GPRIN3
INO80	PMAIP1	RNF38	SPATS2L
RTN4	HMGA2	TSHZ1	RPSAP58
CCT6A	UBE2W	EREG	C5orf60
B4GALT5	MTFMT	RAI2	TPD52
TMED7	TAF13	NFIA	C7orf55

DCP2	ARPC3	SKIDA1	KIF2B
MXD1	GPR52	SRSF2	TMEM47
RAB34	TEX26	ZBTB18	CKMT1A
ITGA9	SLC38A2	IGF1	AGO4
PRNP	REEP3	WDR20	GPC6
SZRD1	BLOC1S2	FICD	DLEU7
HOMER1	BOD1L2	C7orf60	SNCG
DDX6	FRAT2	HABP4	WDR61
MMD	NAP1L5	FRZB	ZHX1
MRAS	TOB1	MMGT1	GREM2
BARHL2	FAM98B	SH3D19	CPA6
OTX2	NUS1	GJA1	PTCHD3
ZDHHC7	LOXL2	VPS37A	OR52E8
PNRC2	RDH14	HPRT1	AP006621.5
CCDC144A	ZBTB37	TMEM55A	SH2D4B
PIGA	KLHL42	PPARG	CST3
MAFB	SLC25A16	CALM2	RP11-664D7.4
ST8SIA3	UBE2G1	CCDC126	LCN15
WDR47	COMMD8	DDX6	OR4M1
STX3	PRKCD	ZMAT3	DUS1L
RNF219	MSMO1	CD69	PPP2R3C
WDR20	CHAC1	ESR1	PNPLA2
SNX3	ARPP21	RAP2C	CDC14A
PTPRA	RHOQ	PMEPA1	MIF4GD
TNRC6A	ACBD5	TSC1	TWF1
AGO4	ZDHHC20	BTG1	KRTAP20-2
C15orf27	OSBPL11	KCNJ10	ZNRF2
UBAP2L	RHOU	PIGA	UNC80
LYSMD2	ADM	TES	GFOD1
EOGT	IPPK	C4orf36	PTEN
MMP15	WBSCR16	PURG	PRKCE
ARRDC3	GPALPP1	TROVE2	AGO1
CTSA	SMAD1	LDLR	PATE4
USP48	TMEM106B	ULK2	AC068987.1
CDC14A	RGS4	RRAGD	HTR2A
ARFIP1	MARCH11	CBFB	ERLIN1
MTMR9	STRBP	TGFBR2	B3GALT4
KAT7	TTC13	HS3ST5	HELLS
ZNF274	SKP2	CHMP3	NUDCD2
PPP1R10	LRRC2	HBP1	RPL34
BAI3	CCDC28A	ZNF217	NRIP1
ABCB7	ANKS1B	RNF103-CHMP3	CDKN1C
MDM4	SNN	NPNT	BST1
SPRYD7	CDC6	HOXB1	PDCD10
PPP6R1	TM4SF18	ZNF3	ARHGAP17
C1GALT1	ZNF410	KALRN	LDOC1
CCDC6	GNPNAT1	MIER1	SNURF
CABP7	DDX52	FOSL1	ATP13A4
PHAX	BOD1	MID1IP1	EIF5
SNX27	ZFC3H1	CPEB3	AXIN2
SOS1	PTEN	ACBD5	LAPTM4A
SMIM12	NABP1	ARHGAP12	PTPLB
C7orf60	FAM214B	C9orf69	PPP6C
NRAS	ARMCX2	ST18	TK2
FBXO28	STK39	CDK19	RDH11
NR2C2AP	CDK8	SPG20	RRAGC
SESTD1	MNX1	UBC	KIAA0247
FEZ2	RNF141	MEOX2	VAPB
NPTX1	ACSL3	TRERF1	AC007461.1
NCKIPSD	EPC1	COX8C	CD80
DDAH1	ADAM9	SLC2A4RG	TMEM221
CDK19	RCN2	FAM175B	NAA60
ELAVL4	ASPN	FNDC4	RP11-826N14.2
KLF6	DYRK1A	PTEN	JAKMIP2
ERRFI1	MINPP1	KCNA4	AMMECR1
VMP1	ATF2	MTMR6	EPHA3
FXR1	SYT10	DSEL	SNX12
SMS	ACTR3	SLC6A6	RET
BICC1	DCDC2	C5orf30	KRTAP20-1
EIF4E3	GBP3	LONRF1	PHF19
MARCH3	RCN1	SNAP25	CDK5R1
MTF1	SHC4	CPEB2	SNX3
KLF5	EIF4G2	KLF3	RPUSD1
PNPLA6	HOXA5	HIVEP2	SH3BP4
C3orf58	CKS2	ATP2B2	C14orf105
TGFA	MTM1	QKI	RP11-1026M7.2
UCP3	PFDN4	EPC2	SMPD1

Supplemental table s4. GO Term of Biological Process

Term	Count	p value	Genes
GO:0045449~regulation of transcription	66	0.007	SLC2A4RG, EZH2, PPARG, MITF, RHOQ, CBFB, ZBTB37, EPC1, RNF141, S1PR1, MIER1, MED26, FOSL1, ZHX1, OTX2, BARHL2, ESR1, CDK8, HMGA2, MXD1, TRERF1, HMGA1, ZNF3, NRIP1, TAF13, MTF1, KDM2A, EREG, CD80, BTG1, UBC, MNX1, ST18, MDM4, ACVR1, ZNF274, TSHZ1, MYBL1, ATF2, NPAS3, BLOC1S2, HOXA5, HBP1, HELLS, KLF5, POLR3G, MAF, KLF6, KLF7, CREBZF, MAFB, IGF1, SMAD1, RPS6KA5, CDKN1C, HOXB1, ZNF217, PHF19, MEOX2, PNRC2, IRF1, HIVEP2, HABP4, NFIA, KLF3, ZNF410
GO:0006350~transcription	55	0.008	SLC2A4RG, EZH2, PPARG, MITF, CBFB, ZBTB37, EPC1, MIER1, MED26, FOSL1, ZHX1, BARHL2, CDK8, ESR1, HMGA2, MXD1, TRERF1, HMGA1, ZNF3, NRIP1, TAF13, MTF1, EREG, KDM2A, MNX1, ST18, ZNF274, TSHZ1, MYBL1, RRAGC, ATF2, TROVE2, NPAS3, HOXA5, HBP1, HELLS, POLR3G, MAF, KLF5, KLF6, KLF7, CREBZF, MAFB, PPP1R10, SMAD1, HOXB1, ZNF217, PHF19, PNRC2, IRF1, HIVEP2, HABP4, NFIA, ZNF410, KLF3
GO:0006355~regulation of transcription, DNA-dependent	47	0.013	ZNF274, SLC2A4RG, TSHZ1, MITF, PPARG, EZH2, RHOQ, MYBL1, CBFB, ATF2, EPC1, RNF141, NPAS3, S1PR1, HOXA5, MED26, FOSL1, HELLS, POLR3G, MAF, KLF5, KLF6, KLF7, CREBZF, MAFB, ZHX1, BARHL2, OTX2, ESR1, IGF1, SMAD1, HMGA2, HMGA1, TRERF1, ZNF3, NRIP1, RPS6KA5, CDKN1C, HOXB1, MTF1, MEOX2, MNX1, IRF1, HIVEP2, MDM4, ST18, NFIA
GO:0051252~regulation of RNA metabolic process	47	0.018	ZNF274, SLC2A4RG, TSHZ1, MITF, PPARG, EZH2, RHOQ, MYBL1, CBFB, ATF2, EPC1, RNF141, NPAS3, S1PR1, HOXA5, MED26, FOSL1, HELLS, POLR3G, MAF, KLF5, KLF6, KLF7, CREBZF, MAFB, ZHX1, BARHL2, OTX2, ESR1, IGF1, SMAD1, HMGA2, HMGA1, TRERF1, ZNF3, NRIP1, RPS6KA5, CDKN1C, HOXB1, MTF1, MEOX2, MNX1, IRF1, HIVEP2, MDM4, ST18, NFIA
GO:0007242~intracellular signaling cascade	36	0.011	PRKAG3, MRAS, RHOQ, RHOU, RRAGC, TMED7, TRIAP1, S1PR1, SOS1, SOS2, TGFA, DDAH1, SHC4, ADAM9, RET, RAP2C, ESR1, DCDC2, IGF1, SMAD1, STRADB, PRKCE, HOMER1, PRKCD, NRIP1, RPS6KA5, NRAS, CD80, ADM, RGS4, RAB34, CKS2, ARL8B, PMEPA1, KALRN, HTR2A
GO:0010604~positive regulation of macromolecule metabolic process	30	0.001	MITF, PPARG, RHOQ, GJA1, CBFB, EPC1, S1PR1, BLOC1S2, FOSL1, ADAM9, MAF, KLF5, KLF6, KLF7, MAFB, BARHL2, ESR1, IGF1, SMAD1, HMGA1, TRERF1, NRIP1, HOXB1, MTF1, EREG, CD80, UBC, IRF1, SH3D19, ACVR1
GO:0006793~phosphorus metabolic process	30	0.009	CDK19, PPP6C, CDK5R1, CDC14A, PTEN, MTM1, TGFA, STK39, MTMR9, MTMR6, ADAM9, MINPP1, RET, TWF1, PAN3, TGFBR2, PTPRA, CDK8, NEK10, STRADB, PRKCE, PRKCD, EPHA3, RPS6KA5, SBF2, ULK2, DYRK1A, IPPK, KALRN, ACVR1
GO:0006796~phosphate metabolic process	30	0.009	CDK19, PPP6C, CDK5R1, CDC14A, PTEN, MTM1, TGFA, STK39, MTMR9, MTMR6, ADAM9, MINPP1, RET, TWF1, PAN3, TGFBR2, PTPRA, CDK8, NEK10, STRADB, PRKCE, PRKCD, EPHA3, RPS6KA5, SBF2, ULK2, DYRK1A, IPPK, KALRN, ACVR1
GO:0031328~positive regulation of cellular biosynthetic process	28	0.000	PPARG, MITF, RHOQ, CBFB, EPC1, S1PR1, BLOC1S2, DDAH1, FOSL1, MAF, KLF5, KLF6, KLF7, MAFB, BARHL2, ARPP19, IGF1, SMAD1, HMGA1, TRERF1, NRIP1, HOXB1, EREG, CD80, MTF1, UBC, IRF1, ACVR1
GO:0009891~positive regulation of biosynthetic process	28	0.000	PPARG, MITF, RHOQ, CBFB, EPC1, S1PR1, BLOC1S2, DDAH1, FOSL1, MAF, KLF5, KLF6, KLF7, MAFB, BARHL2, ARPP19, IGF1, SMAD1, HMGA1, TRERF1, NRIP1, HOXB1, EREG, CD80, MTF1, UBC, IRF1, ACVR1
GO:0051173~positive regulation of nitrogen compound metabolic process	27	0.000	PPARG, MITF, RHOQ, HPRT1, CBFB, EPC1, S1PR1, BLOC1S2, DDAH1, FOSL1, MAF, KLF5, KLF6, KLF7, MAFB, IGF1, SMAD1, HMGA1, TRERF1, NRIP1, HOXB1, EREG, CD80, MTF1, UBC, IRF1, ACVR1
GO:0042127~regulation of cell proliferation	27	0.003	PPARG, MITF, FGFRL1, GJA1, PTEN, S1PR1, BLOC1S2, TGFA, AXIN2, FOSL1, KLF5, CDC6, TGFBR2, IGF1, SMAD1, CDKN1C, NRAS, EREG, TSC1, CD80, ADM, BTG1, MDM4, MAB21L1, LDOC1, TOB1, HTR2A
GO:0010557~positive regulation of macromolecule biosynthetic process	26	0.001	PPARG, MITF, RHOQ, CBFB, EPC1, S1PR1, BLOC1S2, FOSL1, MAF, KLF5, KLF6, KLF7, MAFB, BARHL2, IGF1, SMAD1, HMGA1, TRERF1, NRIP1, HOXB1, EREG, CD80, MTF1, UBC, IRF1, ACVR1
GO:0010628~positive regulation of gene expression	25	0.000	KLF5, MAF, KLF6, KLF7, MAFB, MITF, PPARG, ESR1, IGF1, RHOQ, SMAD1, HMGA1, TRERF1, CBFB, NRIP1, EPC1, HOXB1, S1PR1, MTF1, BLOC1S2, CD80, UBC, IRF1, FOSL1, ACVR1
GO:0045935~positive regulation of nucleobase, nucleoside, nucleotide and nucleic acid metabolic process	25	0.001	KLF5, MAF, KLF6, KLF7, MAFB, MITF, PPARG, IGF1, RHOQ, SMAD1, HMGA1, TRERF1, CBFB, NRIP1, EPC1, HOXB1, S1PR1, MTF1, BLOC1S2, CD80, EREG, UBC, IRF1, FOSL1, ACVR1
GO:0008219~cell death	25	0.004	BID, RTN4, PDCD10, VAPB, ZMAT3, MMD, GJA1, PMAIP1, HPRT1, PTEN, PNPLA6, RRAGC, FXR1, TRIAP1, EIF4G2, NRAS, SOS1, SOS2, SPG20, UBC, MDM4, AXIN2, GADD45A, HTR2A, KALRN
GO:0016265~death	25	0.005	BID, RTN4, PDCD10, VAPB, ZMAT3, MMD, GJA1, PMAIP1, HPRT1, PTEN, PNPLA6, RRAGC, FXR1, TRIAP1, EIF4G2, NRAS, SOS1, SOS2, SPG20, UBC, MDM4, AXIN2, GADD45A, HTR2A, KALRN
GO:0042981~regulation of apoptosis	25	0.016	BID, RTN4, CDK5R1, ZMAT3, MITF, SKP2, ESR1, IGF1, PMAIP1, STRADB, PRKCE, PTEN, NRAS, TRIAP1, BLOC1S2, BTG1, SOS1, SOS2, UBC, PRNP, FOSL1, HELLS, KALRN, ACVR1, ADAM9
GO:0043067~regulation of programmed cell death	25	0.018	BID, RTN4, CDK5R1, ZMAT3, MITF, SKP2, ESR1, IGF1, PMAIP1, STRADB, PRKCE, PTEN, NRAS, TRIAP1, BLOC1S2, BTG1, SOS1, SOS2, UBC, PRNP, FOSL1, HELLS, KALRN, ACVR1, ADAM9
GO:0010941~regulation of cell death	25	0.018	BID, RTN4, CDK5R1, ZMAT3, MITF, SKP2, ESR1, IGF1, PMAIP1, STRADB, PRKCE, PTEN, NRAS, TRIAP1, BLOC1S2, BTG1, SOS1, SOS2, UBC, PRNP, FOSL1, HELLS, KALRN, ACVR1, ADAM9
GO:0045941~positive regulation of transcription	24	0.000	KLF5, MAF, KLF6, KLF7, MAFB, PPARG, MITF, IGF1, RHOQ, SMAD1, TRERF1, HMGA1, CBFB, NRIP1, EPC1, HOXB1, S1PR1, CD80, BLOC1S2, MTF1, UBC, IRF1, FOSL1, ACVR1
GO:0006357~regulation of transcription from RNA polymerase II promoter	23	0.018	MAF, KLF6, KLF7, MAFB, PPARG, MITF, BARHL2, IGF1, RHOQ, SMAD1, CBFB, NRIP1, CDKN1C, EPC1, HOXB1, S1PR1, MTF1, MED26, IRF1, MNX1, MDM4, ST18, FOSL1
GO:0006468~protein amino acid phosphorylation	21	0.025	CDK19, RET, TWF1, CDK5R1, PAN3, TGFBR2, PTPRA, CDK8, NEK10, STRADB, PRKCE, PRKCD, EPHA3, RPS6KA5, ULK2, DYRK1A, TGFA, STK39, KALRN, ADAM9, ACVR1
GO:0030182~neuron differentiation	20	0.001	KLF7, RET, CDK5R1, STX3, OTX2, BARHL2, GJA1, HPRT1, PTEN, CDKN1C, ATP2B2, S1PR1, ADM, DYNLL2, ULK2, UBC, MNX1, SNAP25, FEZ2, KALRN
GO:0045893~positive regulation of transcription, DNA-dependent	19	0.004	MAF, KLF6, KLF7, MAFB, PPARG, MITF, IGF1, RHOQ, SMAD1, TRERF1, HMGA1, CBFB, NRIP1, EPC1, HOXB1, S1PR1, MTF1, IRF1, FOSL1
GO:0051254~positive regulation of RNA metabolic process	19	0.004	MAF, KLF6, KLF7, MAFB, PPARG, MITF, IGF1, RHOQ, SMAD1, TRERF1, HMGA1, CBFB, NRIP1, EPC1, HOXB1, S1PR1, MTF1, IRF1, FOSL1
GO:0042325~regulation of phosphorylation	18	0.007	CDC6, CDK5R1, TGFBR2, IGF1, STRADB, PRKCE, PRKCD, PTEN, CDKN1C, CD80, TSC1, EREG, RGS4, CKS2, TGFA, GADD45A, HTR2A, ADAM9
GO:0051174~regulation of phosphorus metabolic process	18	0.010	CDC6, CDK5R1, TGFBR2, IGF1, STRADB, PRKCE, PRKCD, PTEN, CDKN1C, CD80, TSC1, EREG, RGS4, CKS2, TGFA, GADD45A, HTR2A, ADAM9
GO:0019220~regulation of phosphate metabolic process	18	0.010	CDC6, CDK5R1, TGFBR2, IGF1, STRADB, PRKCE, PRKCD, PTEN, CDKN1C, CD80, TSC1, EREG, RGS4, CKS2, TGFA, GADD45A, HTR2A, ADAM9
GO:0048666~neuron development	17	0.001	KLF7, RET, CDK5R1, STX3, OTX2, GJA1, HPRT1, PTEN, CDKN1C, ATP2B2, ADM, DYNLL2, ULK2, UBC, SNAP25, FEZ2, KALRN
GO:0008285~negative regulation of cell proliferation	17	0.001	CDC6, FGFRL1, PPARG, TGFBR2, GJA1, SMAD1, PTEN, CDKN1C, ADM, TSC1, EREG, BTG1, MDM4, AXIN2, LDOC1, FOSL1, TOB1
GO:0032268~regulation of cellular protein metabolic	17	0.017	EIF4E3, CPEB2, EIF5, BARHL2, IGF1, CPEB1, PRKCE, PRKCD, EIF4G2, CD80, TSC1, UBC, QKI, SH3D19, MIF4GD, TNRC6A, ADAM9

process			
GO:0030030~cell projection organization	16	0.004	KLF5, KLF7, CDK5R1, STX3, OTX2, GJA1, HPRT1, PTEN, ATP2B2, ADM, TSC1, ULK2, UBC, SNAP25, FEZ2, KALRN
GO:0045944~positive regulation of transcription from RNA polymerase II promoter	16	0.004	MAF, KLF6, MAFB, PPARG, MITF, IGF1, RHOQ, SMAD1, CBFB, NRIP1, EPC1, HOXB1, S1PR1, MTF1, IRF1, FOSL1
GO:0051338~regulation of transferase activity	15	0.010	CDC6, CDK5R1, TGFBR2, PPARG, STRADB, PTEN, CDKN1C, TSC1, EREG, RGS4, CKS2, TGFA, GADD45A, HTR2A, ADAM9
GO:0010608~posttranscriptional regulation of gene expression	14	0.000	EIF4E3, CPEB2, EIF5, BARHL2, CPEB1, SMAD1, PTEN, PRKCD, EIF4G2, TSC1, QKI, MDM4, TNRC6A, MIF4GD
GO:0007167~enzyme linked receptor protein signaling pathway	14	0.012	RET, TGFBR2, PTPRA, RHOQ, SMAD1, GREM2, PTEN, EPHA3, RPS6KA5, EREG, SOS1, ADAM9, TOB1, ACVR1
GO:0045859~regulation of protein kinase activity	14	0.013	CDC6, CDK5R1, TGFBR2, STRADB, PTEN, CDKN1C, TSC1, EREG, RGS4, CKS2, TGFA, GADD45A, HTR2A, ADAM9
GO:0043549~regulation of kinase activity	14	0.017	CDC6, CDK5R1, TGFBR2, STRADB, PTEN, CDKN1C, TSC1, EREG, RGS4, CKS2, TGFA, GADD45A, HTR2A, ADAM9
GO:0032989~cellular component morphogenesis	14	0.036	CDK5R1, KLF7, AGFG1, OTX2, GJA1, STRADB, HPRT1, ATP2B2, ADM, ULK2, UBC, SNAP25, FEZ2, KALRN
GO:0008284~positive regulation of cell proliferation	14	0.047	KLF5, NRAS, S1PR1, EREG, BLOC1S2, CD80, ADM, TGFBR2, TGFA, IGF1, MDM4, MAB21L1, FOSL1, HTR2A
GO:0048514~blood vessel morphogenesis	13	0.001	RTN4, KLF5, TGFBR2, PTEN, PNPLA6, S1PR1, MEOX2, NUS1, EREG, QKI, TGFA, C1GALT1, ACVR1
GO:0001568~blood vessel development	13	0.002	RTN4, KLF5, TGFBR2, PTEN, PNPLA6, S1PR1, MEOX2, NUS1, EREG, QKI, TGFA, C1GALT1, ACVR1
GO:0001944~vasculature development	13	0.003	RTN4, KLF5, TGFBR2, PTEN, PNPLA6, S1PR1, MEOX2, NUS1, EREG, QKI, TGFA, C1GALT1, ACVR1
GO:0031175~neuron projection development	13	0.003	KLF7, CDK5R1, STX3, ADM, ULK2, OTX2, UBC, GJA1, HPRT1, SNAP25, PTEN, FEZ2, KALRN
GO:0000902~cell morphogenesis	13	0.036	ATP2B2, KLF7, CDK5R1, ADM, ULK2, OTX2, UBC, GJA1, STRADB, HPRT1, SNAP25, FEZ2, KALRN
GO:0001525~angiogenesis	12	0.000	KLF5, RTN4, S1PR1, NUS1, MEOX2, EREG, TGFBR2, TGFA, PTEN, C1GALT1, PNPLA6, ACVR1
GO:0010647~positive regulation of cell communication	12	0.045	CDKN1C, TMED7, NRAS, CD80, EREG, MIER1, SOS1, UBC, ESR1, TGFA, GJA1, IGF1
GO:0051726~regulation of cell cycle	12	0.048	CDKN1C, TRIAP1, CDC6, CDK5R1, EREG, SKP2, CKS2, TGFA, IGF1, FOSL1, GADD45A, PTEN
GO:0051960~regulation of nervous system development	11	0.003	RTN4, ACTR3, CDK5R1, BARHL2, PPARG, UBC, NPTN, IGF1, SMAD1, SNAP25, KALRN
GO:0048812~neuron projection morphogenesis	11	0.007	KLF7, CDK5R1, ADM, ULK2, OTX2, UBC, GJA1, HPRT1, SNAP25, FEZ2, KALRN
GO:0045321~leukocyte activation	11	0.016	KLF6, CD80, TGFBR2, IRF1, HPRT1, TPD52, PRKCD, HELLS, CBFB, ZNF3, ADAM9
GO:0048858~cell projection morphogenesis	11	0.017	KLF7, CDK5R1, ADM, ULK2, OTX2, UBC, GJA1, HPRT1, SNAP25, FEZ2, KALRN
GO:0032990~cell part morphogenesis	11	0.022	KLF7, CDK5R1, ADM, ULK2, OTX2, UBC, GJA1, HPRT1, SNAP25, FEZ2, KALRN
GO:0001775~cell activation	11	0.044	KLF6, CD80, TGFBR2, IRF1, HPRT1, TPD52, PRKCD, HELLS, CBFB, ZNF3, ADAM9
GO:0006351~transcription, DNA-dependent	11	0.048	KLF5, MAF, TROVE2, TAF13, ZNF217, EREG, MED26, ESR1, IRF1, FOSL1, CBFB
GO:0006417~regulation of translation	10	0.001	EIF4G2, EIF4E3, CPEB2, TSC1, BARHL2, EIF5, QKI, CPEB1, MIF4GD, TNRC6A
GO:0060284~regulation of cell development	10	0.015	RTN4, ACTR3, CDK5R1, BTG1, BARHL2, PPARG, UBC, NPTN, SMAD1, KALRN
GO:0048667~cell morphogenesis involved in neuron differentiation	10	0.017	ATP2B2, KLF7, CDK5R1, ULK2, OTX2, UBC, HPRT1, SNAP25, FEZ2, KALRN
GO:0000904~cell morphogenesis involved in differentiation	10	0.040	ATP2B2, KLF7, CDK5R1, ULK2, OTX2, UBC, HPRT1, SNAP25, FEZ2, KALRN
GO:0010648~negative regulation of cell communication	10	0.044	TSC1, RGS4, EZH2, AXIN2, FRZB, PRKCD, PTEN, TOB1, HTR2A, ACVR1
GO:0046578~regulation of Ras protein signal transduction	9	0.044	NRAS, FICD, S1PR1, AGFG1, TSC1, SOS1, SOS2, IGF1, KALRN
GO:0007517~muscle organ development	9	0.045	MTM1, TSC1, MEOX2, MRAS, IGF1, HOMER1, PTEN, FXR1, SERP1
GO:0044087~regulation of cellular component biogenesis	8	0.017	ACTR3, S1PR1, TSC1, ARPC3, UBC, RHOQ, SNAP25, PTEN
GO:0016311~dephosphorylation	8	0.026	MTM1, PPP6C, CDC14A, SBF2, PTPRA, MTMR9, MTMR6, PTEN
GO:0050767~regulation of neurogenesis	8	0.037	RTN4, ACTR3, CDK5R1, BARHL2, PPARG, NPTN, SMAD1, KALRN
GO:0009894~regulation of catabolic process	7	0.009	CST3, GJA1, IGF1, PNPLA2, SH3D19, MDM4, ADAM9
GO:0051329~interphase of mitotic cell cycle	7	0.013	CDKN1C, PPP6C, CDC6, SKP2, RHOU, GADD45A, ACVR1
GO:0051325~interphase	7	0.015	CDKN1C, PPP6C, CDC6, SKP2, RHOU, GADD45A, ACVR1
GO:0007568~aging	7	0.017	NRAS, TFRC, ADM, LOXL2, PRKCD, PTEN, HTR2A
GO:0050804~regulation of synaptic transmission	7	0.043	ATP2B2, NRAS, SNCG, UBC, NPTN, PATE4, HTR2A
GO:0000079~regulation of cyclin-dependent protein kinase activity	6	0.003	CDKN1C, CDC6, CDK5R1, CKS2, GADD45A, PTEN
GO:0040029~regulation of gene expression, epigenetic	6	0.015	EPC1, CREBZF, SMAD1, HMGA1, TNRC6A, HELLS

GO:0010959~regulation of metal ion transport	6	0.018	ATP2B2, CACNA2D1, GJA1, HOMER1, CALM2, HTR2A
GO:0031344~regulation of cell projection organization	6	0.026	RTN4, ACTR3, BARHL2, RHOQ, SMAD1, KALRN
GO:0043269~regulation of ion transport	6	0.038	ATP2B2, CACNA2D1, GJA1, HOMER1, CALM2, HTR2A
GO:0007178~transmembrane receptor protein serine/threonine kinase signaling pathway	6	0.044	TGFBR2, SMAD1, GREM2, ADAM9, TOB1, ACVR1
GO:0009896~positive regulation of catabolic process	5	0.013	GJA1, IGF1, PNPLA2, SH3D19, ADAM9
GO:0031329~regulation of cellular catabolic process	5	0.027	CST3, IGF1, PNPLA2, SH3D19, ADAM9
GO:0051924~regulation of calcium ion transport	5	0.040	ATP2B2, CACNA2D1, GJA1, HOMER1, CALM2
GO:0010975~regulation of neuron projection development	5	0.042	RTN4, ACTR3, BARHL2, SMAD1, KALRN
GO:0048661~positive regulation of smooth muscle cell proliferation	4	0.020	S1PR1, EREG, TGFBR2, IGF1
GO:0031331~positive regulation of cellular catabolic process	4	0.029	IGF1, PNPLA2, SH3D19, ADAM9
GO:0051147~regulation of muscle cell differentiation	4	0.036	EREG, BTG1, EZH2, UBC
GO:0045667~regulation of osteoblast differentiation	4	0.046	SMAD1, AXIN2, TOB1, ACVR1
GO:0045742~positive regulation of epidermal growth factor receptor signaling pathway	3	0.017	EREG, SOS1, TGFA
GO:0050773~regulation of dendrite development	3	0.031	ACTR3, SMAD1, KALRN
GO:0010469~regulation of receptor activity	3	0.044	EREG, TGFA, PRKCD

Supplemental table s5. GO Term of Cellular Component

Term	Count	p Value	Genes
GO:0000932~cytoplasmic	5	0.001	DCP2, PNRC2, CPEB1, TNRC6A, DDX6
GO:0005768~endosome	15	0.001	MMGT1, LDLR, SNX27, GNPNAT1, MMD, VPS37A, GJA1, MARCH3,
GO:0030426~growth cone	5	0.014	CDK5R1, STX3, TSC1, OTX2, SNAP25
GO:0030427~site of polarized	5	0.015	CDK5R1, STX3, TSC1, OTX2, SNAP25
GO:0043025~cell soma	8	0.027	RTN4, ATP2B2, SNCG, CDK5R1, AGFG1, SOS1, ESR1, KALRN
GO:0009897~external side of	8	0.029	S1PR1, CD80, ATP6AP2, CD69, GPC6, TGFBR2, MMP15, ADAM9
GO:0043005~neuron projection	12	0.037	ATP2B2, ANKS1B, SNCG, CDK5R1, STX3, TSC1, OTX2, ESR1, CPEB1,
GO:0031225~anchored to	9	0.038	NRAS, ART3, BST1, GPC6, PALM3, RHOQ, PRNP, RHOU, SNAP25
GO:0045202~synapse	12	0.046	ACTR3, ANKS1B, CDK5R1, SYT10, SOS1, NPTN, CPEB1, HOMER1,
